# Aflatoxin biocontrol effectiveness in the real world—Private sector-led efforts to manage aflatoxins in Nigeria through biocontrol-centered strategies

**DOI:** 10.3389/fmicb.2022.977789

**Published:** 2022-09-02

**Authors:** O. T. Ola, O. O. Ogedengbe, T. M. Raji, B. Eze, M. Chama, O. N. Ilori, M. A. Awofisayo, L. Kaptoge, R. Bandyopadhyay, A. Ortega-Beltran, A. A. Ndarubu

**Affiliations:** ^1^Harvestfield Industries Limited, Lagos, Nigeria; ^2^International Institute of Tropical Agriculture, Ibadan, Nigeria

**Keywords:** aflatoxin, maize, biocontrol, field management, effectiveness

## Abstract

Aflatoxins are toxic compounds produced by several *Aspergillus* species that contaminate various crops. The impact of aflatoxin on the health of humans and livestock is a concern across the globe. Income, trade, and development sectors are affected as well. There are several technologies to prevent aflatoxin contamination but there are difficulties in having farmers use them. In Nigeria, an aflatoxin biocontrol product containing atoxigenic isolates of *A. flavus* has been registered with regulatory authorities and is now being produced at scale by the private company Harvestfield Industries Limited (HIL). The current study reports results of biocontrol effectiveness trials in maize conducted by HIL during 2020 in several locations across Nigeria and compared to untreated maize from nearby locations. Also, maize was collected from open markets to assess levels of contamination. All treated maize met tolerance thresholds (i.e., <4 ppb total aflatoxin). In contrast, most maize from untreated fields had a higher risk of aflatoxin contamination, with some areas averaging 38.5 ppb total aflatoxin. Maize from open markets had aflatoxin above tolerance thresholds with even an average of up to 90.3 ppb. Results from the trials were presented in a National Workshop attended by key officers of Government agencies, farmer organizations, the private sector, NGOs, and donors. Overall, we report (i) efforts spearheaded by the private sector to have aflatoxin management strategies used at scale in Nigeria, and (ii) deliberations of key stakeholders to ensure the safety of crops produced in Nigeria for the benefit of farmers, consumers, and industries.

## Introduction

Aflatoxins are highly toxic compounds that contaminate several crops including maize, groundnut, and sorghum, and various products derived from them (e.g., livestock feed, peanut butter; Cotty et al., 1994; Iqbal et al., 2010; Magamba et al., 2017). Several *Aspergillus* species can produce aflatoxins, however, *A. flavus* is the most common causal agent of contamination and therefore of global interest (Klich, 2007). Aflatoxin-producers contaminate crops in the field when there are favorable environmental conditions, a susceptible host, and damage by insects, among other factors (Widstrom, 1979; Williams, 2006; Mehl et al., 2012). The problem can become worse after harvest if crops are improperly stored (Grenier et al., 2014; Waliyar et al., 2015). Consumption of crops contaminated with unsafe aflatoxin concentrations can lead to serious health impacts including liver cancer, immunosuppression, stunted growth, and sometimes death (Coursaget et al., 1993; Gieseker, 2004; Gong et al., 2008; Leroy et al., 2018; Voth-Gaeddert et al., 2018). This has resulted in the establishment and enforcement of stringent regulations, especially in developed countries (Cheli et al., 2014). Similar regulations exist in various developing countries, including Nigeria, Ghana, and Malawi; however, these are not properly enforced and, therefore, populations are chronically exposed to the toxins (Matumba et al., 2017; Chilaka et al., 2022).

Apart from health impacts, the contamination seriously affects farmers by limiting their trade opportunities in domestic, regional, and international premium markets, thereby reducing their income (Williams, 2008; Wu, 2015). Because of health, trade, and income impacts, it is crucial to use technologies to reduce aflatoxin contamination across the value chain. The use of biocontrol products containing atoxigenic (i.e., non-aflatoxin-producing) isolates of *A. flavus* allows farmers to produce crops with significantly less aflatoxin compared to non-treated crops (Mehl et al., 2012). This technology was developed for use in the United States (Dorner, 2004; Cotty et al., 2007) and has been adapted, improved, and registered for use in several countries in Africa, including Nigeria (Bandyopadhyay et al., 2016, 2022). However, although highly effective in African countries, there are challenges to developing and implementing sustainable programs to have biocontrol used at scale.

In Nigeria, the first aflatoxin biocontrol product outside the US was developed by an initiative co-led by the International Institute of Tropical Agriculture (IITA) and the United States Department of Agriculture—Agricultural Research Service (USDA-ARS) and various local and international partners (Atehnkeng et al., 2016; Bandyopadhyay et al., 2016). This product, named Aflasafe™, was developed, tested, registered, and used by farmers across Nigeria in efforts primarily led by IITA (Bandyopadhyay et al., 2019). Farmers in about 100,000 ha were incentivized to use Aflasafe across Nigeria in efforts facilitated by IITA and it was demonstrated that aflatoxin management using biocontrol-centric approaches was possible through policy support and market incentives (Bandyopadhyay et al., 2022). However, producing and using biocontrol at scale, and sustainably, necessitated transferring the technology to the private sector. IITA and partners developed a well-planned commercialization strategy that allowed the understanding of (i) market segments, (ii) projected demand, (iii) manufacturing economics, (iv) break-even scenarios, and (v) mapping potential private sector manufacturing and/or distribution partners. These steps finally allowed shortlisting of the most appropriate investors (Konlambigue et al., 2020). In March 2018, through a competitive process, IITA granted the company Harvestfield Industries Limited (HIL) the license to manufacture and distribute Aflasafe in Nigeria (Bandyopadhyay et al., 2022).

In both 2018 and 2019, IITA backstopped HIL from the manufacturing, distribution, and use of the biocontrol product across Nigeria. Then, in 2020, HIL completely took over those responsibilities to make the transition to reduce reliance on IITA. The current study reports (i) commercial usage of the biocontrol product through innovative approaches in efforts completely led by HIL, (ii) aflatoxin content in maize collected from fields treated with biocontrol, untreated fields, and open markets in selected areas of all six geopolitical regions of Nigeria, and (iii) evidence that private-sector led efforts can result in mobilization of resources for effective aflatoxin management for safer food in Nigeria and elsewhere.

## Materials and methods

### Biocontrol product manufacturing

The biocontrol product Aflasafe used in the 2020 maize planting season was produced by HIL in the Aflasafe Demonstration Manufacturing Plant in IITA-Ibadan under a toll manufacturing agreement. In 2020, the HIL manufacturing plant was under construction and has been completed in December 2021. Briefly, the industrial process includes coating roasted, sterile sorghum grain with a spore suspension of each of four atoxigenic isolates of *A. flavus*, the active ingredients of the product (Atehnkeng et al., 2016). The spores are coated on the sterile grains with the aid of a polymer. A blue food colorant is added to differentiate the product from regular sorghum grain. The industrial process and the quality tests have been described in great detail (Bandyopadhyay et al., 2016).

### HIL efforts for effective aflatoxin management

**Figure 1:**
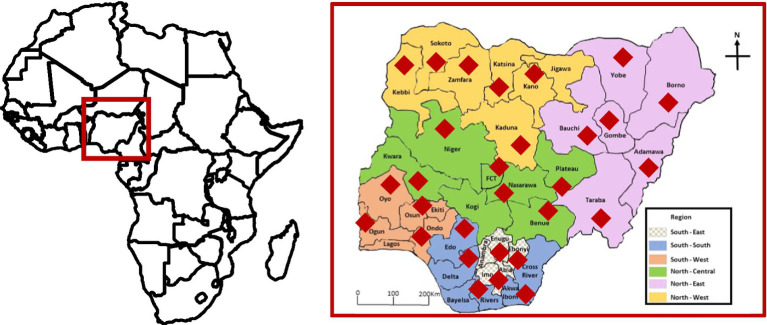
Map of Africa (left) showing the location of Nigeria and map of Nigeria (right) showing its six geopolitical zones. The biocontrol product was used in several states in each zone. The red diamonds show the states where (i) the biocontrol effectiveness trials in maize were established and (ii) where maize from open markets were sampled.

HIL negotiated with the Central Bank of Nigeria-Anchor Borrowers’ Programme (CBN-ABP) to include Aflasafe in the list of inputs for farmers. Established by the Central Bank of Nigeria in 2015, CBN-ABP aims to promote local food production, through the provision of locally produced inputs to farmers. Through the Maize Growers, Processors, and Marketers Association of Nigeria (MAGPAMAN), Aflasafe was distributed to farmers across Nigeria (Figure 1). In parallel, HIL engaged with food quality regulatory agencies on enforcing aflatoxin testing across the value chain starting at the aggregation centers, and provided aflatoxin-testing services across the country.

### Farmer selection and training

Farmers and farmer-field selections were carried out in collaboration with the lead farmers from MAGPAMAN and Agricultural Extension Agents (AEAs) of the Agricultural Development Project (ADP). Lead farmers and AEAs were trained by HIL in April 2020 on aflatoxin contamination and its management using good agronomic practices, and pre-and post-harvest interventions, including the use of biocontrol. Then, lead farmers and AEAs passed the knowledge to the farmers that they oversee. Trainings were conducted in villages, or at facilities of Local Government Areas (LGAs).

### Field treatment

Biocontrol application was conducted in fields across the six geopolitical regions of Nigeria during the 2020 cropping season, which spans from April to October. Maize farmers belonging to MAGPAMAN were trained on aflatoxin contamination and its field management by lead farmers and AEAs as above. In the current study, we report results from selected locations in each of the six geopolitical regions (Figure 1). In general, fields that were selected for the study were ploughed, harrowed, and ridged before planting maize. Farmers followed the MAGPAMAN recommendations for their areas and used pre-planting, pre-emergence, and post-emergence herbicides. Most farmers also used insecticides (e.g., Emamectin benzoate 1 l/ha) to control Fall Armyworm. Biocontrol-treated and untreated fields that were paired for the comparisons were separated by at least 500 m to prevent biocontrol interference.

In treated fields, the biocontrol product was broadcast by hand 30 to 45 days after planting, depending on the variety planted, at the rate of 10 kg/ha as described before (Bandyopadhyay et al., 2019). Farmers were advised to avoid field operations 7-to-14 days after treatment to prevent trampling on the product. Lead farmers and AEAs monitored biocontrol treatment, sporulation, and general operations of the farmers. In untreated fields, farmers conducted field operations only following the general recommendations of MAGPAMAN.

### Training to conduct *in-situ* aflatoxin quantification

In September 2020, IITA trained around 30 HIL staff stationed across the country on crop sampling and the use of equipment to quantify aflatoxins in the field and stored crops. The training included practical demonstrations on the use of the GIPSA-approved Neogene Raptor Reader and Neogen Reveal Q+ for Aflatoxin kit (Neogen Corp., Lansing, MI). The knowledge obtained by HIL staff allowed them to conduct the sampling and aflatoxin quantification on their own. Before this, HIL invested in eight units (scanner, kits, reference materials, pipettes, tips, filter papers, and blender, among other materials) to conduct the quantification in the selected locations across Nigeria.

### Sample collection and *in-situ* aflatoxin quantification

Maize ears were randomly harvested by hand from treated and untreated fields by farmers and AEAs under the supervision of HIL staff. The ears were placed in bags and transported to nearby locations where *in-situ* aflatoxin quantification analyses were carried out (e.g., the house of the lead farmer or aggregation point). For the maize that was already harvested and stored (~1 week) by farmers, treated and untreated grains were randomly collected from bags stacked in farmers’ warehouses. About 1 kg was collected per sample, placed in plastic bags, labeled accordingly, and immediately used for the *in-situ* aflatoxin quantification analyses in the presence of the farmers and AEAs. Maize samples were also obtained from the open markets within each of the selected localities and the same *in-situ* analysis was conducted on the same day of sampling. About 1 kg of maize was randomly collected from multiple bags of the maize offered by the vendors.

Briefly, each sample was blended into a powder. The blender was washed with 80% ethanol between samples to prevent cross-contamination. A 20 g sub-sample of milled maize was combined with 100 ml 65% ethanol and blended for 1 min. The mixture was then filtered through Whatman No. 1 filter paper (Whatman Intl. Ltd., Maidstone, England) into a 100 ml beaker. Thereafter, 500 μl of sample diluent was measured into a sample cup and 100 μl of sample filtrate was added and mixed thoroughly. Finally, 400 μl of the aliquot of the diluted sample was transferred to the cartridge of the Reveal® Q+ Aflatoxin Reader Raptor to measure the aflatoxin concentration.

### Data analysis

For the biocontrol treatment comparisons, means were separated using paired Student *t*-tests (α = 0.05). Before the analysis, aflatoxin concentration data (*x*) were transformed using the equation *y* = log10(1 + *x*) to stabilize the variance. Statistical tests were conducted with SAS version 9.2 (SAS Institute Inc., Cary, NC). Percent reduction was calculated for each region as follows: [(mean aflatoxin content of untreated maize – mean aflatoxin content of Aflasafe-treated maize)/mean aflatoxin content of untreated maize] × 100.

### Presentation of results

HIL in collaboration with IITA held a workshop in April 2021 to (i) share results of aflatoxin levels in treated and untreated maize under the CBN-ABP 2020 wet season project, and (ii) pledge concerted efforts towards the adoption of appropriate technologies for the reduction of aflatoxin in crops, foods, and feeds as required by global food quality standards.

## Results

### Number of farmers participating in the commercial trials

Over 73,000 maize farmers across Nigeria belonging to MAGPAMAN were trained by lead farmers and AEAs as above on aflatoxin contamination and its field management. The average field size of each farmer was 1.5 ha.

### Aflatoxin content in maize from biocontrol-treated and untreated fields

**Figure 2:**
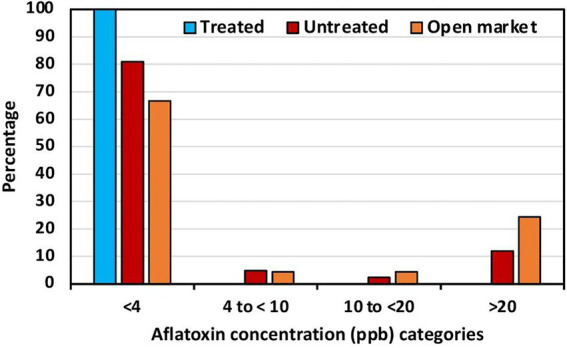
Percentage of maize from biocontrol treated fields, untreated fields, and open market in each of four total aflatoxin concentration categories.

Aflatoxin levels in maize in selected locations across the six geopolitical regions in Nigeria are shown in Table 1. Low aflatoxin levels were detected in all maize from treated fields (range = 0.2 to 3.8 ppb). Untreated maize from North West (NW) and South–South (SS) contained low aflatoxin levels (max = 3.7 ppb). However, in the other four regions, the average aflatoxin content in untreated maize at harvest ranged from 7.6 ppb in North East (NE) to 38.5 ppb in South East (SE; Table 1). There were some maize samples greatly exceeding international standards, with up to 168 ppb detected in a sample from SE. The variance in maize from treated fields was very low, never exceeding 1, while in untreated maize the variance reached up to 3,470 (Table 1). The average aflatoxin content in maize from treated fields was always lower than in maize from untreated fields (Table 1). All the treated maize samples had less than 4 ppb total aflatoxin and consequently none in the other categories (Figure 2). About 80% of the untreated maize had less than 4 ppb, about 8% between 4 and 20 ppb categories, and ~12% had over 20 ppb (Figure 2). Aflatoxin reductions in maize from treated fields ranged from 8.7% to 97.4% less aflatoxin than corresponding untreated maize. Significantly less aflatoxin content occurred in four of the six regions where the comparisons were conducted (Table 1).

**Table 1:** Aflatoxin concentration in harvested maize from biocontrol treated, untreated, and untreated (open market) in Nigeria.

Region^a^	Treatment^b^	*N*	Aflatoxin concentration (ppb)^c^				% Reduction^d^
			Min	Max	Mean	Variance	
NW	Treated	21	0.4	3.5	1.9	0.8	9.5
	Untreated	25	0.7	3.4	2.1	0.5	
	Open market	10	0.4	175.5	19.7	2,697.6	
NE	Treated	14	1.0	3.1	1.8	0.3	76.3*
	Untreated	24	0.7	89.9	7.6	359.1	
	Open market	5	2.5	225.9	90.3	9,586.3	
NC	Treated	16	0.9	3.2	1.7	0.4	94.5*
	Untreated	18	1.1	140.5	30.8	2,223.6	
	Open market	7	1.3	77.9	15.3	681.9	
SW	Treated	9	1.6	3.7	2.9	0.4	82.9*
	Untreated	6	2.3	86.3	17.0	961.8	
	Open market	8	1.4	180.2	42.5	4,843.9	
SE	Treated	6	0.2	1.6	1.0	0.2	97.4*
	Untreated	6	3.0	167.6	38.5	3,470.1	
	Open market	8	1.3	219.1	53.0	6,172.9	
SS	Treated	10	1.0	3.8	2.1	0.7	8.7
	Untreated	5	1.5	3.7	2.3	0.6	
	Open market	7	1.4	132.1	22.2	2,025.0	

a NW, North West; NE, North East, NC, North Central; SW, South West; SE, South East; SS, South South.

b Treated refers to fields to which the biocontrol product was applied at the rate of 10 kg/ha. Untreated were nearby fields separated by at least 500 m from corresponding treated fields in which no biocontrol product was applied. Open market refers to untreated maize purchased in informal markets in nearby locations where the trials were conducted.

c ppb, parts per billion.

d Percent reduction was calculated as: [(mean aflatoxin content of untreated maize – mean aflatoxin content of Aflasafe-treated maize)/mean aflatoxin content of untreated maize] × 100. An asterisk (*) indicates significant (*p* < 0.05) differences in aflatoxin content between treated and untreated maize in each region (Student’s *t*-test; α = 0.05).

### Aflatoxin content in maize from the open market

The average aflatoxin content in maize from the open market ranged from 15.3 ppb in North Central (NC) to 90.3 ppb in NE (Table 1). In all regions, except NC, there were samples with well over 100 ppb, and even over 200 ppb in both NE and SE. The variance ranged from 681 in NC to 9,686 in NE (Table 1). Around 25% of the maize from the open market had >20 ppb total aflatoxin (Figure 2).

### Workshop

**Figure 3:**
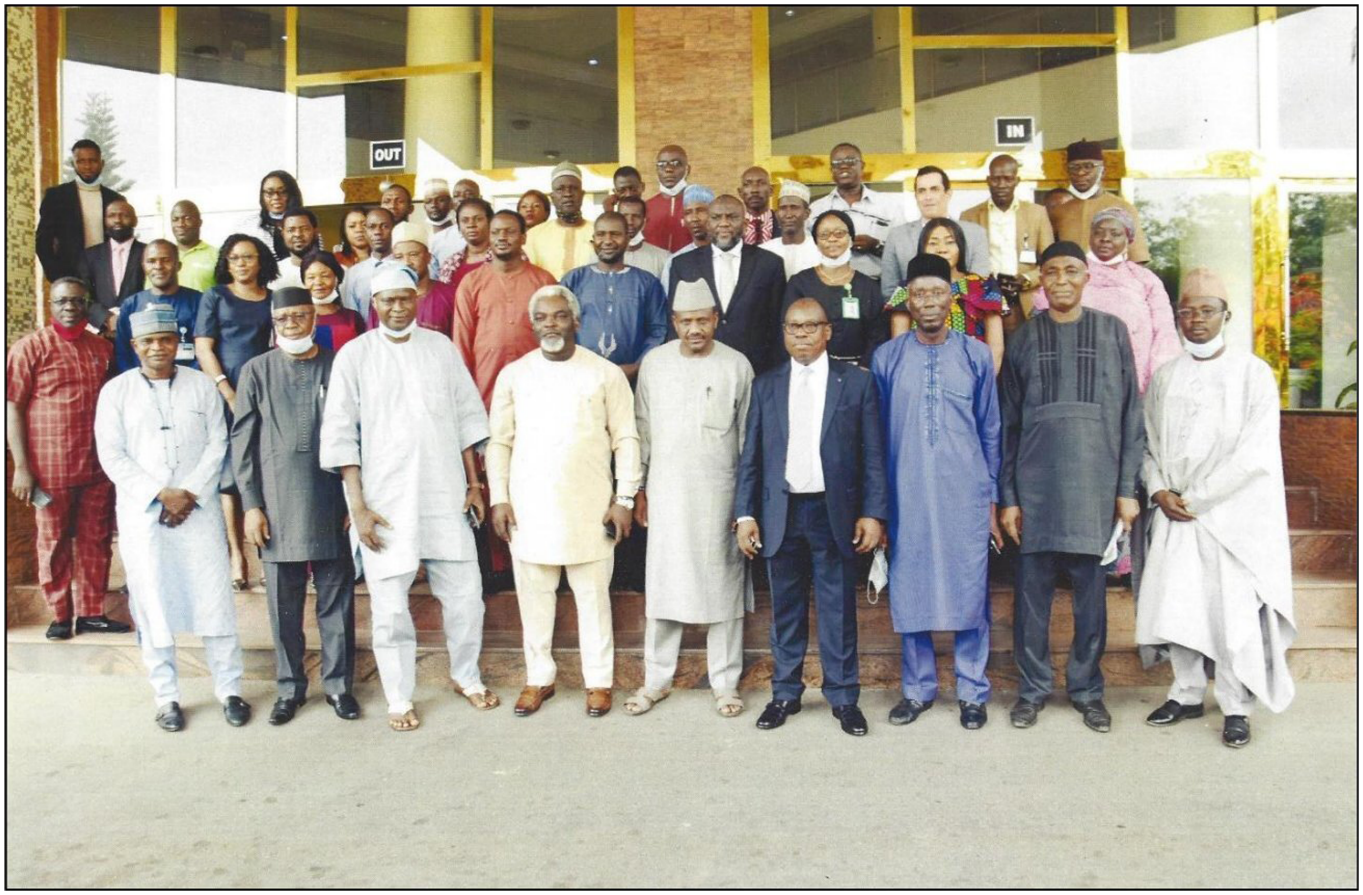
Participants of the National Workshop in Abuja, Nigeria. The objective of the workshop was to (i) discuss the effectiveness commercial trials during the CBN-ABP 2020 wet season project, and (ii) discuss the way forward to converge efforts of the different institutions to mitigate aflatoxins in Nigeria.

Participants at the workshop (Figure 3) included representatives of the Central Bank of Nigeria (CBN), Federal Ministry of Agriculture and Rural Development (FMARD), National Agency for Food and Drug Administration and Control (NAFDAC), Standards Organization of Nigeria (SON), Federal Competition and Consumer Protection Commission (FCCPC), Federal Ministry of Health (FMOH), Value Seeds Ltd., Maize Association of Nigeria (MAAN), MAGPAMAN, National Groundnut Producers Processors and Marketers Association of Nigeria (NGROPPMAN), National Sesame Seed Association of Nigeria (NSSAN), National Ginger Association of Nigeria (NGAN), Ginger Growers, Processors and Marketers Association of Nigeria (GGPMAN), Sorghum Farmers Association of Nigeria (SOFAN), National Association of Sorghum Producers, Processors and Marketers of Nigeria (NASPPAM), Hybrid Feeds, Poultry Association of Nigeria, National Agricultural Extension and Research Liaison Services (NAERLS), Technoserve Intl., Global Alliance for Improved Nutrition (GAIN), Flour Mills of Nigeria (FMN), One Acre Fund, Palm Valley Ltd., Alluvial Agriculture Ltd., Olam Intl., Babban Gona Agriculture Services, Coopetition Forum for Aflatoxin-Reduced Agricultural Products (CFARAP), Nestle, Women Farmers Advancement Network (WOFAN), and Winrock International.

Apart from discussing the results of the effectiveness of biocontrol treatment in the selected locations, participants at the workshop discussed several topics and agreed on the following key recommendations:

1. The re-establishment of the inter-ministerial committee on aflatoxin regulation and enforcement of food safety laws in Nigeria.
2. Enactment of technical policies regulating the testing and enforcement of allowable aflatoxin limits in food and feed processing and distribution industries.
3. Capacity development and provision of infrastructural testing facilities at relevant grain collection centers.
4. Empowerment of relevant agencies with the necessary quality standard and procedure for testing aflatoxin contamination and the authority to ensure individuals and organizations comply with approved aflatoxin limits.
5. Constitute a Secretariat and Working Group to coordinate the “Scaling Up of Aflatoxin Solutions in Nigeria,” with each relevant participant nominating a high-level representative.

## Discussion

The current study provides additional evidence that the use of the biocontrol product Aflasafe significantly reduces aflatoxin in maize when compared to maize from untreated fields in various locations across Nigeria. Although the effectiveness of the product during testing and commercial use has been reported before in Nigeria through efforts led by a research institution (Atehnkeng et al., 2016; Bandyopadhyay et al., 2019), this is the first time in Nigeria in which effectiveness is reported in efforts led entirely by a private sector company, HIL. The company HIL relied on IITA only for the training for sampling crops to conduct the comparisons and the use of an aflatoxin quantification system to conduct analyses *in-situ*. Results from the current study demonstrate that aflatoxin management can be sustainably and independently promoted and scaled up by the private sector following an initial phase of technical backstopping by researchers. Also, we report high-level engagement efforts to properly address the aflatoxin contamination crises in Nigeria. Overall, challenges in implementing a research concept for the benefit of farmers, consumers, and the population, in general, can be overcome when mobilizing key stakeholders in agricultural value chains: farmers, scientists, regulators, private sector, government officers, consumers, extension agents, among others.

Aflatoxin biocontrol offers a solution for a problem that cannot be seen and can only be detected through chemical analyses. The biocontrol technology is, therefore, and unfortunately, not appreciated by the market making the technology difficult to promote. This difficulty applies to any other aflatoxin management technology. IITA demonstrated that manufacturing the technology at scale was possible (Bandyopadhyay et al., 2016), but the commercialization part requires other sets of skills, finances, connections, and infrastructure (Konlambigue et al., 2020). This is an area in which HIL excels having a deep understanding of the agricultural situation in Nigeria, well-established contacts, and a distribution network across the country. Therefore, IITA selected HIL due to its (i) wide reach in the farming community, (ii) willingness to invest in a new manufacturing plant, (iii) excellent relationships with different government sectors, and (iv) social responsibility to make significant efforts to push a technology to solve a problem that cannot be seen but that has large impacts on health, trade, and income.

Because of the difficulties of marketing the biocontrol product (for example, it is comparatively easier to sell a fertilizer or a product that controls fall armyworm), it was necessary to place efforts to conduct product value demonstration activities together with the end-users. The best way to demonstrate the value of the product is by conducting *in-situ* analyses instead of collecting samples to be analyzed in laboratories far away from the fields, as demonstrated in The Gambia (Senghor et al., 2021). The *in-situ* testing assay was used in the trials of the current study. The assay increases confidence among farmers, AEAs, and other stakeholders in the aflatoxin management system centered on biocontrol that farmers used. This assay is critical for manufacturers and distributors to demonstrate to farmers and interested industries both the value of implementing an integrated aflatoxin management system and the risk of producing crops with unsafe aflatoxin levels if the system is not adopted.

All maize samples from the treated fields had low aflatoxin levels and qualified for local and international aflatoxin-conscious markets (Table 1; Figure 2). Aflatoxin content variance in treated maize was always very low (range = 0.2 to 0.8) while in the untreated maize it reached up to 3,470 (Table 1). Reduced aflatoxin variance in treated crops indicates a reduced risk of aflatoxin contamination (Senghor et al., 2020). On the other hand, although not all untreated maize was contaminated, the high variance indicates that there is a high risk of the untreated maize being contaminated at harvest. Similarly, there was a high variance in aflatoxin content in maize from the open markets (up to 9,586; Table 1). Maize, groundnut, and chili pepper treated in Nigeria with the same biocontrol product have proven to be effective (Atehnkeng et al., 2014; Bandyopadhyay et al., 2019; Ezekiel et al., 2019). The use of country-specific biocontrol products has similarly resulted in low aflatoxin levels in treated crops in other countries (Alaniz Zanon et al., 2016; Mauro et al., 2018; Savić et al., 2020; Senghor et al., 2020, 2021; Ortega-Beltran et al., 2021). However, previous trials in Nigeria were part of research efforts to determine whether the product was effective, the correct dosage, the best ways to manufacture the product, determine the willingness of farmers to use the product, build mechanisms to incentivize farmers to use the product, and generate evidence to showcase to the interested private and public sector to invest in the technology. After technology transfer, this is the first time in which the effectiveness of biocontrol usage in Nigeria is reported for trials conducted by the private sector.

Not all untreated maize contained a high aflatoxin concentration. In two of the four regions, NW and SS, the average aflatoxin content in untreated maize was only 8%–9% higher than that in the treated maize, but still without surpassing the strictest threshold of 4 ppb (Table 1). This could be attributed to fungal communities with low or no aflatoxin production potential in the treated fields or unfavorable conditions for aflatoxin production. However, in other trials, maize from NW and SS areas has been reported to contain up to 300 ppb (Bandyopadhyay et al., 2019). This is an example of the uncertainty of aflatoxin contamination with some years resulting in low aflatoxin levels while in others in extremely high aflatoxin content. When untreated maize has relatively low aflatoxin, the biocontrol application may result in reduced calculated effectiveness (i.e., only 8%–9% in NW and SS; Table 1). The greatest beneficial effect of biocontrol is revealed when there is high aflatoxin pressure in the untreated crops.

High aflatoxin concentrations in maize from the open market, including in both NW and SS, were detected (Table 1). There were some samples with extremely high aflatoxin content. Results of the current study demonstrate that although oftentimes untreated maize at harvest contain low aflatoxin levels, once offered in local markets the contamination can significantly increase due to improper handling. Biocontrol-treated crops have been reported to contain low aflatoxin levels after 3–6 months of storage even under sub-optimal conditions (Bandyopadhyay et al., 2019; Senghor et al., 2020). Other studies report high aflatoxin content in maize in SW, with an average of 142 ppb (Akande et al., 2017), while Ayeni et al. (2020) also reported an average aflatoxin content of 126 ppb from several maize samples obtained in major markets. Keta et al. (2019) reported high content of aflatoxin in millet (range = 41 to 58 ppb) and maize (range = 12 to 57 ppb) collected in open markets in the Northern region of Nigeria. If such crops sold in open markets had been treated with biocontrol and other management practices were used as well, the aflatoxin concentration would have been significantly lower, and consumers could have been less exposed to the dangerous toxins.

Apart from the effectiveness of the product, another positive outcome of the commercial trials was the evidence of appropriate training on correct product usage. Correct product usage was demonstrated because all treated maize contained safe aflatoxin levels (Table 1; Figure 2). Lack of proper training impedes attaining maximum aflatoxin biocontrol efficacy (Hoffmann et al., 2018). Farmers need to become familiar with the product and become aware, appreciate, and enjoy the benefits of protecting their crops using biocontrol and other management strategies. Farmers, technicians, and extensionists need to spend considerable time as a team in training sessions, on the field during the application, on follow-up visits, and at harvest to ensure aflatoxin management is correctly implemented. In Kenya, the adoption of the biocontrol technology was tested in a simulated market premium for a food safety study. The use of the technology was explained in a half a day training session; the testing scenario did not include extensive training (Hoffmann et al., 2018). Multiple training sessions are needed to attain familiarity with any product for appropriate technology usage and uptake. Manufacturing and distribution licensees of aflatoxin biocontrol must deliver appropriate training and continuous field support to groups of farmers buying the product. Failure to do so would result in incorrect product usage and most likely deficient aflatoxin reductions, which ultimately would damage the reputation of the technology and would reduce its sales.

The National Workshop organized by HIL stimulated the dialogue about the need for nationwide efforts to decrease aflatoxin contamination across crop value chains. Stakeholders from various organizations with distinct mandates actively participated in the meeting to reach a common ground, plan concerted efforts, and avoid working in silos. It is known that strategies to deal with agricultural problems with complex spillover negative impacts have greater chances of positive outcomes when the input of diverse stakeholders (farmers’ organizations, consumers, governments, regulators, donors, scientists, and industries) is incorporated into the planning and implementation (Evans et al., 2020). The participants agreed that there is a need to effectively pursue the key recommendations from the workshop with the aim to, among others (i) establish a policy for testing aflatoxin levels in food and feed commodities in Nigeria, (ii) document aflatoxin allowable limits in Nigerian food quality standards, and (iii) enforce that the food quality standard is enshrined in the regulatory law in Nigeria.

The Aflasafe technology was transferred to HIL through a Technology Transfer and Licensing Agreement (TTLA). This legal document allows one organization (i.e., HIL) to obtain a license to use a patented technology or intellectual property developed by another organization (i.e., IITA). The TTLA allows IITA to receive a small amount of licensing fees and reinvest them into future research products and innovations while also technically backstopping the manufacturer (when requested) to ensure that the product is accessible and successfully scaled. For Aflasafe products, there is a balance to allow private sector investors to make a profit without making it unduly expensive for smallholder farmers to access the product. In 2022 alone, HIL will produce (in their new facility) and sell around 2,000 tons of Aflasafe to protect crops in 200,000 ha. Following the successful 2020 season working with MAGPAMAN growers, facilitated by the CBN-ABP, HIL is currently engaging with the partners for the expansion of the program. Aflasafe has been officially included in the expanded program.

Although there is always room for improvement, there are several lines of denialism about the value of and/or need for aflatoxin biocontrol products for use in Africa. First, there was the notion that biocontrol will not be effective in African environments. It was thought impossible to have products registered with authorities due to the absence of an appropriate regulatory framework. There were also claims about the lack of need for protecting crops (Pitt, 2019), and also that few farmers will afford and adopt the technology (Stepman, 2018; Pitt, 2019). Another criticism is that biocontrol has been targeted for use in a few crops while there are over 50 susceptible crops grown in African nations (Stepman, 2018). Here we report efforts to overcome several putative barriers preventing the sustainable use of biocontrol. There are true challenges to having the product accepted, making it available at scale, developing mechanisms for farmers to buy it, having it correctly used, demonstrating its value, and linking farmers to buyers of aflatoxin-safe crops. However, mechanisms to have biocontrol sustainably used at scale, and converged with other complementary technologies have been developed. A multi-disciplinary IITA-led team composed of scientists, engineers, social scientists, development professionals, communication experts, extension agencies, policymakers, regulators, and business development specialists, among others laid the foundation for the sustainable use of aflatoxin biocontrol in Nigeria. The initial groundwork by IITA and partners has helped HIL sustainably commercialize biocontrol with its manufacturing and distribution capabilities. In addition, HIL judiciously navigated through complex issues such as political stability, regional conflicts, infrastructure, communication campaigns, procurement of materials for manufacturing (thousands of tons of sorghum, bags, polymer, dye), timely production and distribution, correct training of farmers, COVID-19 restrictions, local insecurity, among other factors. Reduced aflatoxin prevalence can result through these efforts and can contribute tremendously toward achieving zero rejection of export commodities and ensuring food safety of crops consumed in Nigeria.

## Conclusion

Although challenging, aflatoxin contamination and exposure are preventable using an integrated field-to-fork strategy converging policy, infrastructure, and technical options, including aflatoxin biocontrol. Here we report that it is possible to have research products implemented for practical use by smallholder farmers in African countries despite political, infrastructural, cultural, climatic, and agricultural challenges. Significant achievements reported here include (i) the notable uptake of the first generation of non-seed CGIAR technologies successfully transcending from the laboratory to the industry for the benefit of farmers and consumers at scale; (ii) stimulation of commercial usage of biocontrol product through innovative approaches (iii) evidence that private-sector-led efforts can result in mobilization of resources for effective aflatoxin management for safer food in Nigeria and elsewhere; and (iv) use of biocontrol for the production of hundreds of thousands of tons of safe maize and groundnut across Nigeria. Finally, there are many areas of active research for the aflatoxin biocontrol technology that deserve to be investigated. These include the development of cheaper, improved formulations, the use of biocontrol products in susceptible crops that have received less attention (e.g., millets, sunflower), and convergence with other practices that decrease aflatoxin contamination. With improved, refined strategies, aflatoxin management programs will surely result in reduced aflatoxin contamination and exposure in Nigeria and elsewhere.

## Data availability statement

The raw data supporting the conclusions of this article will be made available by the authors, without undue reservation.

## Author contributions

OTO, OOO, TR, BE, MC, OI, MA, and AN contributed to the conception and design of the experiments, conducted the training of farmers and extension agents, conducted the field studies, collected samples, conducted aflatoxin quantification, and analyzed the data. LK, RB, AO-B, backstopped HIL staff during the production of Aflasafe and designed the trainings given to HIL for sampling and aflatoxin quantification. OTO, OOO, TR, BE, MC, OI, MA, AO-B, and AN organized the workshop to present the results. OTO, AO-B, and AN drafted the manuscript. All authors read, reviewed, and approved the final version of the manuscript.

## Conflict of interest

LK, RB, and AO-B are employed by IITA. OTO, OOO, TR, BE, MC, OI, and AN were employed by Harvestfield Industries Limited, the company that commercially manufactures and distributes Aflasafe. MA owns stocks of the company.

## Publisher’s note

All claims expressed in this article are solely those of the authors and do not necessarily represent those of their affiliated organizations, or those of the publisher, the editors and the reviewers. Any product that may be evaluated in this article, or claim that may be made by its manufacturer, is not guaranteed or endorsed by the publisher.

## Author disclaimer

Authors associated with IITA receive no direct financial benefit from the manufacturing and marketing of the aflatoxin biocontrol product mentioned in this article. The Aflasafe name is a Trademark of IITA. In the past, IITA used to manufacture and commercialize Aflasafe for use in Nigeria, Senegal, Burkina Faso, The Gambia, and Ghana. Manufacturing and distribution responsibilities have been licensed to the private or public sector. IITA charges a small licensing fee to manufacturers for use of the Aflasafe name and the cost associated with technology transfer and technical backstopping.
